# mRNA Fragmentation Pattern Detected by SHAPE

**DOI:** 10.3390/cimb46090610

**Published:** 2024-09-16

**Authors:** Shanshan Feng, Ting Chen, Yunlong Zhang, Changrui Lu

**Affiliations:** College of Biological Science and Medical Engineering, Donghua University, Shanghai 201620, China; sandyfss@163.com (S.F.); chenting@dhu.edu.cn (T.C.); zhyl@dhu.edu.cn (Y.Z.)

**Keywords:** mRNA stability, degradation, mRNA structure, freeze–thaw cycles, long-term storage

## Abstract

The success of messenger RNA (mRNA) vaccines in controlling COVID-19 has warranted further developments in new technology. Currently, their quality control process largely relies on low-resolution electrophoresis for detecting chain breaks. Here, we present an approach using multi-primer reverse transcription sequencing (MPRT-seq) to identify degradation fragments in mRNA products. Using this in-house-made mRNA containing two antigens and untranslated regions (UTRs), we analyzed the mRNA completeness and degradation pattern at a nucleotide resolution. We then analyzed the sensitive base sequence and its correlation with the secondary structure. Our MPRT-seq mapping shows that certain sequences on the 5′ of bulge–stem–loop structures can result in preferential chain breaks. Our results agree with commonly used capillary electrophoresis (CE) integrity analysis but at a much higher resolution, and can improve mRNA stability by providing information to remove sensitive structures or sequences in the mRNA sequence design.

## 1. Introduction

Since the outbreak of the COVID-19 pandemic and its rapid global spread, mRNA vaccine platforms have emerged as promising strategies for virus infection protection [1,2,3]. These vaccines elicit innate immune responses, safeguarding against viral infections and even virus-induced cancers [4,5,6,7]. However, the inherent instability of mRNA necessitates measures to prevent degradation during storage and transportation. The canonical approach to preserving mRNA integrity involves maintaining low or ultra-low temperatures throughout the entire process. This includes storage at 2–8 °C (refrigeration), −20 °C, or −80 °C (freezing), as well as cold-chain shipping using dry ice or liquid nitrogen [8,9,10,11,12,13]. Unfortunately, such temperature-dependent methods are equipment-intensive, costly and susceptible to failures.

As a solution, mRNA degradation primarily results from the spontaneous cleavage of phosphodiester linkage via transesterification. During this process, a deprotonated 2′-hydroxyl group attacks the phosphate group, forming a pentacoordinate transition state. Consequently, the 5′-oxyanion spontaneously departs, yielding a 2′,3′-cyclic phosphate [14,15]. Therefore, the process requires flexibility in the RNA backbone, where highly structured mRNA can enhance the mRNA functional half-life [16]. For example, base pairing within mRNA shields the backbone’s 2′-hydroxyl group [17,18]; hence, double-strand helices will enhance mRNA stability. In addition, Watson–Crick G-C base pairs (with three hydrogen bonds) are more stable than A-U or other wobble base pairs. However, GC-rich mRNA can raise the difficulty of production, and vaccine platforms often employ mRNA with sizes of several thousand nucleotides that are prone to degradation problems. Understanding the structure–degradation pattern correlation can benefit mRNA design by eliminating potential cleavage sites in mRNA.

Here, we describe multi-primer reverse transcription sequencing (MPRT-seq) as a method for mapping degradation-sensitive sequences using selective 2′-hydroxyl acylation analyzed by primer extension (SHAPE) chemistry. This method was validated on an in-house designed model mRNA carrying a fusion protein payload complete with untranslated regions (UTRs) sequences. This study also examined degradation-sensitive sequences and their corresponding secondary structures. Our MPRT-seq results correlated with capillary electrophoresis integrity analysis. Unstable sequences were mapped to the 5′ side of hairpin stems featuring a top loop and a bulge in the middle stem. This well-established and reliable method can pioneer mRNA Quality Assurance/Quality Control (QA/QC) so that the industry can adopt them without too much trouble.

## 2. Materials and Methods

### 2.1. RNA Synthesis and Secondary Structure Prediction

We designed and synthesized mRNA containing two typical genes: green fluorescent protein (GFP) and luciferase (Luc) flanked by commonly used 5′ and 3′ UTRs. The mRNA was dissolved in RNase-free double-distilled water at a concentration of 2 mg/mL. To predict the secondary structure of GFP-Luc mRNA, we utilized the RNAfold web server that was based on the ViennaRNA Package Version 2.6.3 (http://rna.tbi.univie.ac.at/cgi-bin/RNAWebSuite/RNAfold.cgi, accessed on 30 May 2024.), covering domains from the 5′ UTR to the 3′ UTR. The poly (A) tail did not form base pairs with other mRNA elements.

### 2.2. Simulated mRNA Degradation

To stimulate mRNA degradation, RNA samples were treated two ways: freeze–thaw cycles and 37 °C heating. For freeze–thaw cycles, a 1 mL RNA sample was deposited in a 2 mL tube, the tube bottom was dipped into liquid nitrogen, ensuring that the sample level was below the liquid nitrogen surface. The RNA was soaked for approximately 20 s until the solution was completely frozen. Then, the RNA sample tube bottom was placed in water at room temperature until the solution was fully thawed. This freeze–thaw procedure was repeated as described. Freeze–thaw cycles were repeated 5, 10 and 20 times. After achieving the desired cycles, a 300 μL RNA solution was transferred to a new EP tube. For 37 °C heating, a 600 μL RNA sample deposited in an EP tube was placed in a constant-temperature metal heating block; after 24 h and 48 h, a 300 μL RNA solution for each time was transferred to a new EP tube. An mRNA sample without any treatment from the same batch was used as the control. All the samples were stored in −80 °C for the following detection.

### 2.3. Capillary Electrophoresis (CE) Quality Detection

The 20 μL treated mRNA sample was analyzed using Bioptic Qsep1 CE fragment Analyzer (BiOptic Inc., Taiwan, China) and Cartridge Kit with R1 Cartridge (RNA Cartridge number R1-O-240222-58). The alignment marker was RNA-LM (20 nt). Detection was performed following the manufacturer′s protocol with a total RNA QC program. The CE program analyzed the sample smear and the main peak cutoff, calculated the distribution of the peak area and the average size of the fragment. The analyzed mRNA length ranged from approximately 2100 to 3300 nt, covering the entire main mRNA fragments peak. The result file was reported by the Bioptic Q-Analyzer^®^ software (version 3.4.3). The reported data included the capillary electropherogram, relative fluorescence intensity migration time plot and RNA quality number (RNA fragment range, average fragment length and their distribution). 

### 2.4. MPRT-Seq and Data Analysis

**Figure 1:**
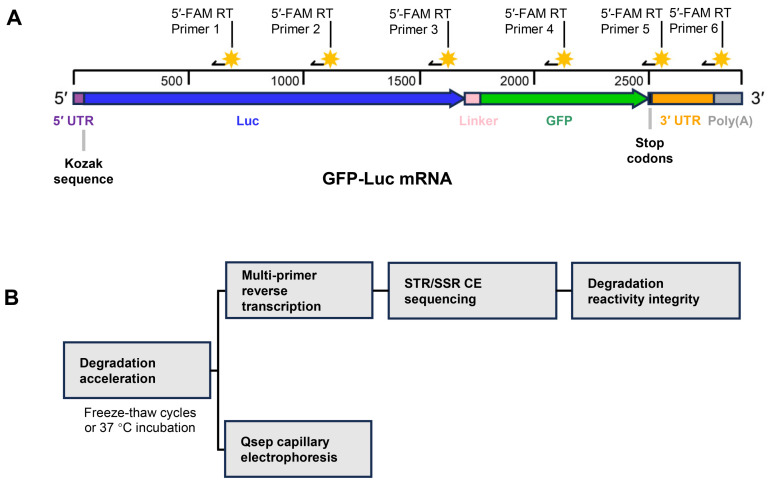
(**A**) Schematic of green fluorescent protein–luciferase (GFP-Luc) mRNA and six reverse-transcription (RT) primer positions. The 5′ untranslated region (UTR) (residues 1–52), Kozak sequence (residues 47–52) at the 3′ end of 5′ UTR, Luc (residues 53–1702), linker (residues 1703–1768), GFP (residues 1769–2485), stop codons (residues 2486–2491), 3′ UTR (residues 2492–2786) and poly (**A**) (residues 2787–2896) are shown in purple, blue, pink, green, black, orange and grey, respectively. The black arrow and the yellow eight-pointed star represent the reverse-transcription DNA primer and 5′-FAM label; (**B**) mRNA degradation study workflow.

SHAPE is a widely used technique for RNA structure analysis [19,20]. mRNA strand breaks due to degradation can also be detected by similar chemistry. However, individual reverse-transcription reads of 300–650 nt typically limit the depth of a single reaction [21]. To further analyze mRNA degradation, an optimized SHAPE method, MPRT-seq, was used. The reverse-transcription (RT) primer pairing positions are shown in Figure 1A. The treated GFP-Luc mRNA RNA was annealed with 6 RT DNA primers, respectively. The 10 μL annealed RNA solution was mixed with the RT assay reagents, including 4 μL of 1 × HiScript III Buffer, 1 μL of 10 mM dNTPs and 10 units of HiScript III Reverse Transcriptase (Vazyme Biotech Co., Ltd., Nanjing, China). The mRNA solution was incubated at 50 °C for 30 min and then quenched with 0.8 μL of 5 M NaOH at 95 °C for 5 min, neutralized by adding 29 μL of an acid stop mix.

The 5′-FAM-labeled DNA oligo sequences are as follow:

RT Primer 1 (pairing position: 554–573): 5′-FAM-GGAGGTAGATGAGATGTGAC-3′;

RT Primer 2 (pairing position: 1036–1053): 5′-FAM-GGCAGATGGAACCTCTTG-3′;

RT Primer 3 (pairing position: 1561–1578): 5′-FAM-GCGGTTGTTACTTGACTG-3′;

RT Primer 4 (pairing position: 2065–2082): 5′-FAM-CCGTCGTCCTTGAAGAAG-3′;

RT Primer 5 (pairing position: 2493–2510): 5′-FAM-CATGCAGTACCAGCTCGA-3′;

RT Primer 6 (pairing position: 2771–2788): 5′-FAM-TTGCTAGCTCCAGGGTGT-3′.

The cDNA products were sequenced by Short Tandem Repeat (STR) CE analysis. Raw data were analyzed using the ShapeFinder Version 1.0 [21] program package. The reactivity was processed in 6 sections led by the 5′-FAM-labeled RT primers; reactivity quantity covered a range of about 500 nt, which was between two adjacent primer-pairing positions, and all the quantity data were assembled, normalized and subtracted by the control reactivity using Excel. An average degradation line was generated according to a rescale equation as follow:

(1) IA(N)=A∗(1+q)^N

where $I_{A}(N)$ is the rescaled average intensity at position *N*, *A* is the total average degradation reactivity value directly obtained from the comprehensive gradient test of freeze–thaw cycles or 37 °C heating, and *q* is the rescale factor.

For the freeze–thaw cycles, the total average reactivity *A* was 3.5 and the rescale factor *q* was 0.000001. For the 37 °C heating, *A* was 8.8 and q was 0.00035. A degradation intensity higher than the relative average value was defined as high intensity. Under this criterion, we identified four sets of sequences, each containing 5 to 10 successive nucleotides, which exhibited high reactivity for both freeze–thaw cycles and 37 °C heating incubation.

## 3. Results

### 3.1. In-Lab mRNA Degradation Acceleration Detected by Both CE and MPRT-Seq

The mRNA design and the study workflow are outlined in the Section 2 and Figure 1B. Specifically, the mRNA sample was treated with freeze–thaw cycles and 37 °C heating, then detected in two ways: (i) Qsep CE and (ii) MPRT-seq, which included multi-primer reverse transcription, Short Tandem Repeat/ Simple Sequence Repeat (STR/SSR) CE sequencing and reactivity integrity.

**Figure 2:**
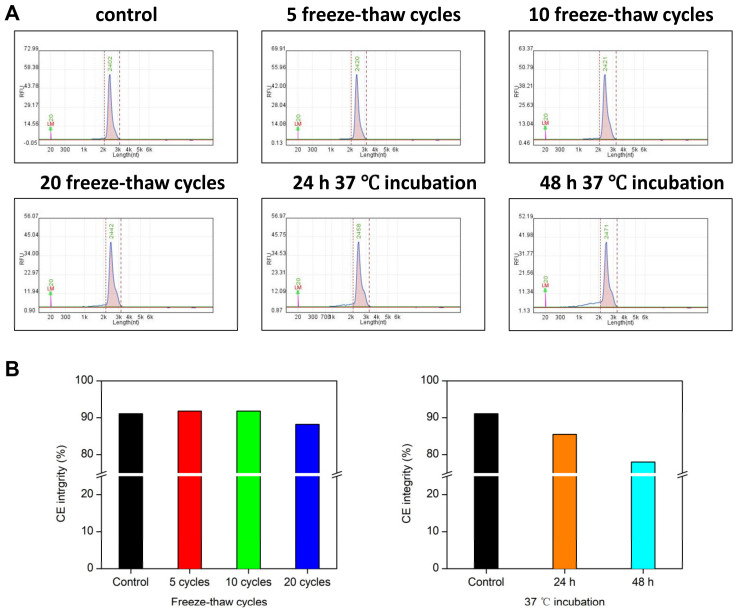
Capillary electrophoresis (CE) evaluation of GFP-Luc mRNA integrity under accelerated degradation. mRNA samples were analyzed using the Qsep CE analyzer. (**A**) Electropherograms of mRNA under accelerated degradation. The freeze–thaw cycles were 5, 10 and 20, respectively; incubation was performed at 37 °C for 24 h and 48 h, respectively. The alignment marker is RNA-LM (20 nt), and dashed lines indicate the cutoff of the main mRNA fragments peak, which ranges from approximately 2100 to 3300 nt. (**B**) mRNA integrity derived from CE analysis: the left panel shows the degradation effect of freeze–thaw cycles, with CE integrity bars for 5, 10 and 20 freeze–thaw cycles shown in red, green and blue, respectively. The right panel shows the degradation effect of 37 °C incubation, with CE integrity bars for 24 h and 48 h incubations shown in orange and cyan, respectively. The CE integrity bar for the control is shown in black.

First, we used standard CE to obtain a baseline for our measurements. Electropherograms in Figure 2A show that the mRNA peak eluted in a length range from approximately 2100 to 3300 nt with an average size about 2500 nt. The control mRNA displays 91.1% integrity, while the mRNA treated with 5 and 10 freeze–thaw cycles both display similar integrity of 91.8%. After 20 cycles, the mRNA sample displays increased integrity loss to 88.2% compared to the control (Figure 2B, left panel, blue bar). On the other hand, 37 °C incubation can degrade our target mRNA more effectively. The CE results show that mRNA integrity dropped to 85.5% after 24 h and 78.0% after 48 h (Figure 2A,B). Using CE analysis, we can determine the general integrity loss of our target mRNA after 37 °C incubation, but less so for the less-harsh freeze–thaw-treated mRNA.

**Figure 3:**
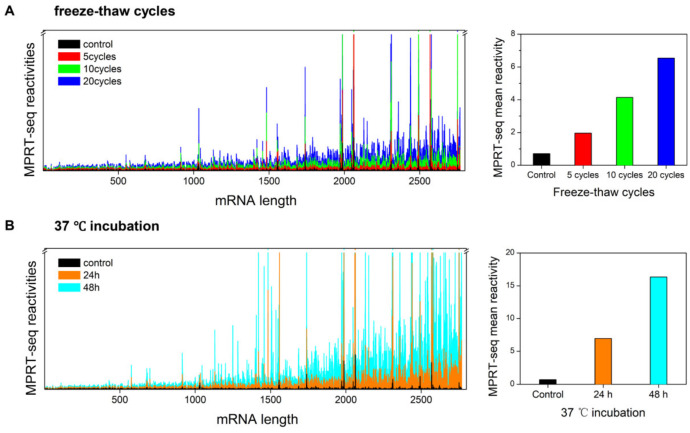
Multi-primer reverse transcription sequencing (MPRT-seq) reveals GFP-Luc mRNA reactivity changes under accelerated degradation. (**A**,**B**) left panels show MPRT-seq reactivity changes under freeze–thaw cycles and 37 °C heating incubation, respectively. mRNA length is plotted on the *X*-axis. The right panels show the mean reactivity of MPRT-seq under degradation acceleration treatment. The coloring of the MPRT-seq mean reactivity bars are consistent with Figure 2.

To investigate the degradation effect on individual sequences and secondary structures more accurately, we employed a multi-primer reverse-transcription sequencing (MPRT-seq) technique using SHAPE chemistry. This method can reliably detect fragments resulting from mRNA chain breaks. Overall, MPRT-seq analysis shows that as freeze–thaw cycles increased from 0 (control) to 20 cycles, we observe a linear increase in average reactivity (Figure 3A, left panel). The average MPRT-seq reactivity raised from 0.71 (control) to 1.95, 4.14 and 6.54 under 5, 10 and 20 freeze–thaw cycles, respectively (Figure 3A, right panel). While the control shows only few small peaks, after freeze–thaw cycles, individual peaks arise corresponding to RNA lengths of 1000, 1500, 2000 and 2500 nt. Peaks around 2000–2500 nt show strongest reactivity (Figure 3A, left panel). Similar to the CE results, MPRT-seq shows that 37 °C heating causes more degradation (Figure 3B, left panel), with the average reactivity increasing to 6.93 and 16.35 (Figure 3B, right panel). Figure 3B shows more intense peaks from the 48 h incubation sample than the 24 h sample. Coincidentally, the 24 h sample produces very similar results to the 20-cycle–freeze-thaw sample (CE: 88.2% vs. 85.5%, MPRT reactivity: 6.93 vs. 6.54). Both samples also shared very similar SHAPE reactivity profiles (Figure 3A,B, blue vs. orange traces).

### 3.2. MPRT-Seq Characterize the Degradation Reactivity at a Single-Nucleotide Resolution

Next, we identified nucleotides with the highest peaks in freeze–thaw cycles and 37 °C heating treatments. These nucleotides are distributed sporadically on the two antigens and the 3′ UTR in a single or sequential manner (Figure 3A,B).

Under freeze–thaw cycles (Figure 3A), in the GFP gene, GCC (1986–1988), CGACC (2308–2312) and UUC (2438–2440) show average increased reactivity of 15.8-, 11.3- and 9.1-fold compared to the corresponding average SHAPE reactivity (3.5). In the 3′ UTR gene, G 2569, G 2577 and U 2751 have increased reactivity 134.6-, 19.6- and 56.9-fold compared to the corresponding average SHAPE reactivity (3.5). The reactivities of nucleotides under freeze–thaw cycles follow a C > G > A ≈ U trend (proportion: 0.48, 0.25, 0.15, 0.12 for C, G, A, U, respectively; total 81 nucleotides).

For 37 °C heating, C 1483 in the luciferase gene has increased reactivity 25.1-fold compared to the average (14.8). In the GFP gene, GCC (1986–1988) increased reactivity 14-fold compared to the corresponding average SHAPE reactivity (17.7), and CCGACC (2307–2312) increased reactivity 8-fold compared to the average (19.8). In the 3′ UTR gene, UAGGU (2574–2578) shows a 9-fold reactivity increase in the average (21.7) and UUGG (2750–2753) shows a 22.1-fold reactivity increase in the average (23.1). On the other hand, under 37 °C heating, the trend of nucleotide reactivities turns to C > G ≈ U > A (proportion: 0.40, 0.29, 0.21, 0.10 for C, G, U, A, respectively; total 94 nucleotides).

### 3.3. Specific Sequences Localized on the Hairpin Stem 5′ Side Sensitive to Degradation

**Figure 4:**
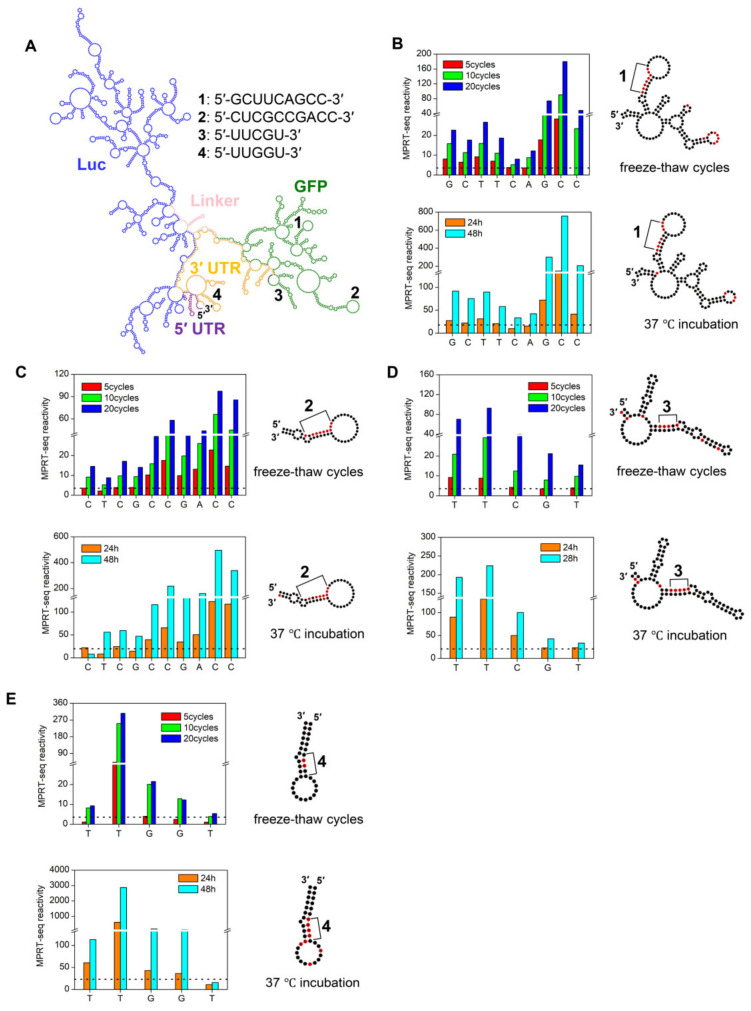
GFP-Luc mRNA secondary structure prediction and evaluation of high-degradation-reactivity sequences. (**A**) Secondary structure prediction was performed using RNAfold web server with the sequence from residue 1 to 2789, except the poly (**A**) tail. The coloring of GFP-Luc mRNA elements is consistent with Figure 1A schematic. Additionally, 1–4 represent sequences that are sensitive to both freeze–thaw cycles and 37 °C heating incubation, and their corresponding positions are shown in the secondary structure. (**B**–**E**) left panels represent MPRT-seq reactivity under degradation acceleration, with consistent secondary structure motifs shown on right side. The dotted line shows the relative mean reactivity derived from Equation (1). The red circles in the secondary structure motifs represent relative high-degradation nucleotide positions, and black circles represent relatively low activities.

To investigate the peaks observed previously, we analyzed secondary structures in these particular locations. The predicted secondary structure of GFP-Luc mRNA (except the poly (A) tail) resembled a trefoil shape (Figure 4A, Figures S1 and S2). Next, we analyzed the relationship between the high reactivity nucleotide locations and their respective secondary structures by mapping them on their predicted secondary structures. We located four sequences with the highest average reactivity nucleotides located on the mRNA sequence; these four sequences are characterized by similar secondary structure and reactivity profiles. Sequence 5′-GCUUCAGCC-3′ (1) is located on 5′ side of a hairpin stem that connects to a four-way junction, and the nucleotides display higher reactivities on base GCC at the 3′ end, and lower reactivities on base CA in the middle (Figure 4B). Sequence 5′-CUCGCCGACC-3′ (2) is located on the 5′ side of an extended hairpin stem, with base CGACC at the 3′ end showing higher reactivity (Figure 4C). Sequence 5′-UUCGU-3′ (3) and 5′-UUGGU-3′ (4) both have similar sequence and reactivity trends, with base UU at the 5′ end showing sharp degradation reactivity (Figure 4D,E). In summary, the corresponding secondary structures of the four sequences located on the 5′ side of the hairpin stems feature a top loop and a bulge in the middle stem.

## 4. Discussion

Understanding mRNA degradation helps in effective mRNA design. In this study, we carried out a series of degradation acceleration procedures and analyzed GFP-Luc mRNA degradation-sensitive sequences and their corresponding secondary structure using CE and MPRT-seq.

Longer mRNA products are more stable under long-term storage conditions in buffers due to more compact tertiary structures. However, longer mRNA presents two significant challenges during production: firstly, a longer mRNA is more difficult to produce and has a lower integrity; secondly, a longer mRNA has a higher potential for internal cleavage under non-buffer storage conditions during mRNA drug production [12,18,22,23]. Although single-GFP or Luc mRNA can also be used in this study, a longer GFP-Luc mRNA may have lower integrity and a higher potential for internal cleavage than single-GFP or Luc mRNA, meanwhile a smaller mRNA cannot represent the actual problems in the mRNA manufacturing industry, where the size of many antigen mRNAs can be up to thousands of nucleotides [6,24,25]. Therefore, we utilized the longest available GFP-Luc fusion mRNA in our laboratory.

Capping and poly (A) tail are critical within mRNA QA/QC. However, many of the said processes warrant improvements and cannot be resolved by a single method. We aimed to improve one aspect of mRNA quality control by combining capillary electrophoresis and RT-PCR. However, this set of methods still has limitations in analyzing the percentages of full-length mRNA with the correct cap and poly (A) tail. Further work to analyze the quality of full-length mRNA with capping and poly (A) tail is needed.

Our results display discrepancies in the degradation reactivity at the nucleotide level, whereby 37 °C samples show higher degradation reactivity. This result is similar with the degradation of hepatitis C virus (HCV) RNA observed by Li et al. [10]. We do not fully understand the mechanism to explain the difference in the rate of degradation. We suspect that during prolonged incubation at 37 °C, any residual RNase or free radicals in the samples may promote mRNA degradation, and the intrinsic spontaneous cleavage activity of the mRNA may also increase.

Our results also show that Luc mRNA is less affected by freeze–thaw cycles. The calculation of MPRT-seq reactivity involves directly averaging all activity values from the comprehensive gradient test of freeze–thaw cycles or 37 °C heating, resulting in a relatively high overall average value. We use this value as a benchmark to identify the nucleotides with a relatively higher degradation intensity. Under these conditions, Luc mRNA near the 5′ end is less affected by degradation compared to the 3′ end GFP and UTR mRNA.

In this study, CE is a quality control method that provides an average of the mRNA population. MPRT-seq analyzes each base and assists in exploring the nucleotides prone to degradation. The decreasing trend in CE integrity is correlated and complementary to the increasing trend in MPRT-seq degradation activity averages (Figure 2 and Figure 3). Further, mapping the MPRT-seq results with the predicted secondary structure, we identified four sequences located at the 5′ side of a loop–stem structure with high degradation activity. Counter-intuitively, they are all located on the 5′ side of a loop–stem structure (Figure 4). Currently, we have no idea why the 5′ stem loop is susceptible to RNA degradation. We hope to resolve this issue in our next manuscript.

There are several other methods for RNA fragment analysis. The most common ones are high-performance liquid chromatography-mass spectrometry/mass spectrometry (HPLC-MS/MS) and microfluidic electrophoresis. HPLC-MS/MS is known for its high sensitivity and specificity, making it suitable for detailed analysis with a high accuracy. However, HPLC-MS/MS is expensive, time-consuming, and requires expertise and equipment [26,27,28]. Microfluidic electrophoresis is quick, automated and low-sample consuming. However, it also requires specific equipment and incurs some consumable costs [29]. In addition to RNA fragment analysis, next- and third-generation sequencing can also be employed for mRNA quality analysis [30]. Next-generation sequencing offers short-to-medium read lengths, which are high-throughput and have high accuracy. Third-generation sequencing, including PacBio and Nanopore, provides real-time sequencing with long or ultra-long read lengths [30,31,32,33,34]. However, they are more expensive and have a higher error rate. Additionally, using next-generation and third-generation sequencing for mRNA quality analysis requires reverse transcription, followed by cDNA PCR or cDNA synthesis base pairing, which may introduce additional bias.

Capillary electrophoresis is currently used as the technique for assuring mRNA integrity due to being quick and easy to use [35,36,37,38]. However, our results show that CE has two major disadvantages that warrant improvements. First, CE has a very limited resolution that does not allow for the screening of pure, complete mRNA. We showed that CE cannot detect any change if small fragments (around 800 nt) break off from the mRNA (Figure 2). Those fragments will most likely contain a truncated antigen, missing its 3′ UTR and poly (A) sequence. Second, CE cannot provide a sequence–degradation correlation because it lacks all sequence information. In this study, we attempted to develop novel methods to resolve these aforementioned problems. Our MPRT-seq result not only agrees well with the CE analysis, but also provides sequence identities for the truncation locations (Figure 3 and Figure 4). Although second- and third-generation sequencing can also achieve sequence specificity, MPRT-seq enables a convenient, accurate and time-efficient analysis of the degree of breakage at the single-nucleotide level. Because of their reliability and wide recognition within the field, the industry can adopt these well-established methods without too much trouble. If any significant degradation location is found, the designer can quickly revise the mRNA sequence and optimize the codons to eliminate a vulnerable site, achieving higher mRNA purity and longer shelf-life, without affecting antigen expression. If enough mRNA MPRT-seq data are accumulated, AI-guided optimizations can also be integrated alongside mRNA optimization algorithms [22,25,39,40,41] to provide a more robust mRNA design protocol. Our primary goal in sequence optimization is to ensure the correct expression of the protein sequence, as sequence specificity is not always critical. We can always change the sequence of RNA using different codons to preserve the target protein sequence. As long as the proteins do no change, some changes in mRNA are almost always tolerated, barring introducing miRNA sites or ribozyme cleavage sites. Secondly, if maintaining sequence specificity is required but the structure is prone to degradation, we can modify the surrounding sequences to complement the target-specific sequence while disrupting the degradation-prone structure. Overall, each case can be addressed individually, as well as building this algorithm into codon optimization software.

In conclusion, our technique utilizes the SHAPE analytical approach to provide high-accuracy quality control for mRNA production. Secondly, MPRT-seq can screen the degradation-sensitive sequences and their structural characteristics. Once developed, this information can provide an mRNA degradation model specific for each mRNA sequence, and guide improved mRNA stability.

## Data Availability

The original contributions presented in this work are included in the article; further inquiries can be directed to the corresponding author.

## Supplementary Materials

The following supporting information can be downloaded at: https://www.mdpi.com/article/10.3390/cimb46090610/s1, Figure S1. Secondary structure prediction of GFP-Luc mRNA and high-degradation-reactivity residual position plot under freeze–thaw cycles. Figure S2. Secondary structure prediction of GFP-Luc mRNA and high-degradation-reactivity residual position plot under 37 °C heating incubation.
