# Supplementary material for: mRNA Fragmentation Pattern Detected by SHAPE
Source: Curr Issues Mol Biol. 2024 Sep 16;46(9):10249–58. doi: 10.3390/cimb46090610 (PMC11431040; doi:10.3390/cimb46090610)
Supplement: Supplementary file 1 [file cimb-46-00610-s001.zip › cimb-3180667-supplementary.pdf]

Supplementary Materials

mRNA Fragmentation Pattern Detected by SHAPE

Shanshan Feng [1], Ting Chen [1], Yunlong Zhang [1] and Changrui Lu [1,*]

[1] College of Biological Science and Medical Engineering, Donghua University, Shanghai 201620, China; sandyfss@163.com (S.F.); chenting@dhu.edu.cn (T.C.); zhyl@dhu.edu.cn (Y.Z.)

[*] Correspondence: Tel: 86-21-67792740. E-mails: crlu@dhu.edu.cn (C.L.)

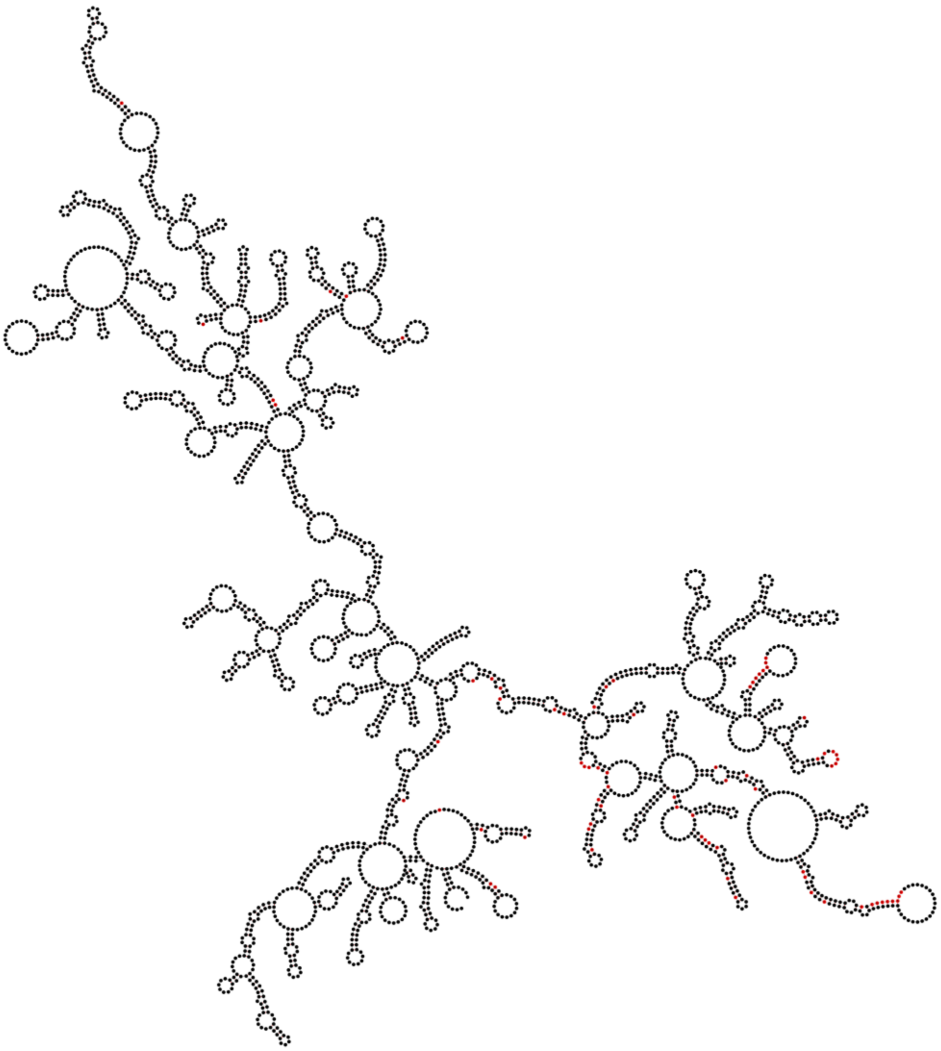

Figure S1. Secondary structure prediction of GFP-Luc mRNA and high-‑degradation-‑reactivity residual positions plot under freeze–-thaw cycles.

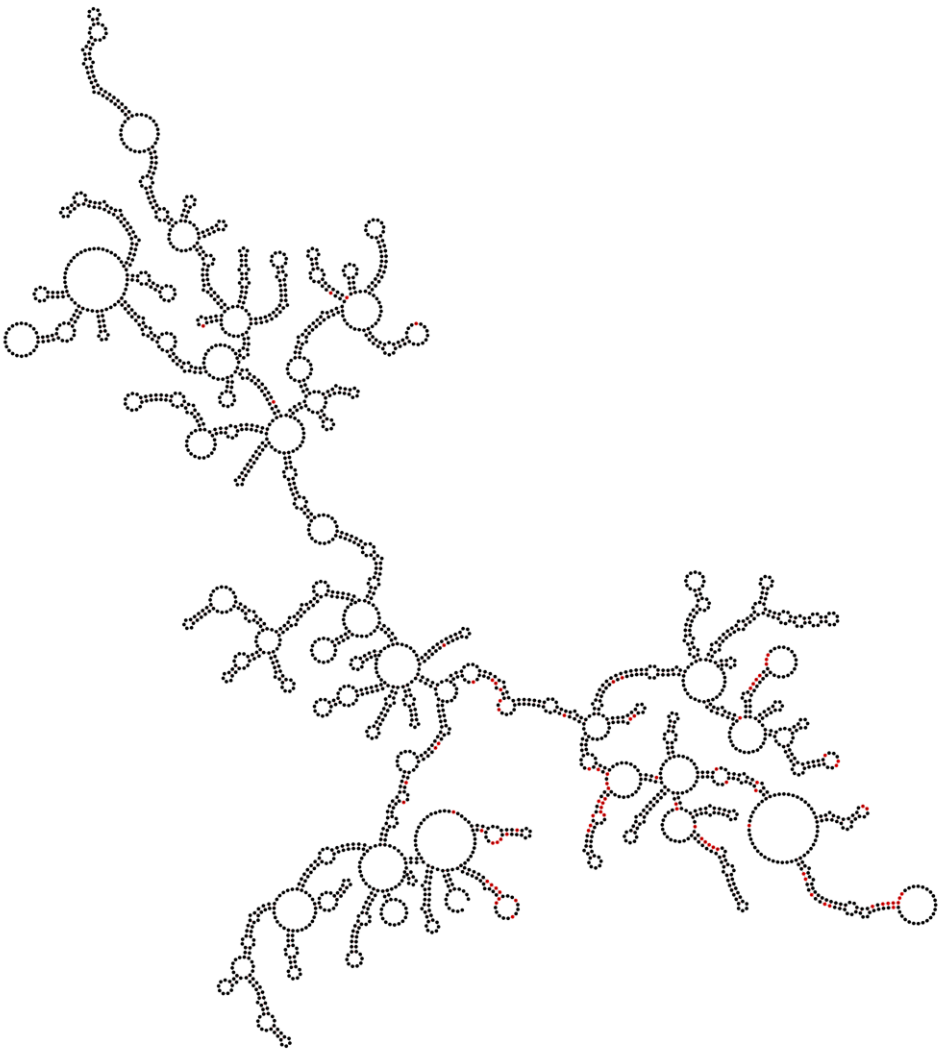

Figure S2. Secondary structure prediction of GFP-Luc mRNA and high-degradation-reactivity residual positions plot under 37 °C heating incubation.
